# Post-Cure Development of the Degree of Conversion and Mechanical Properties of Dual-Curing Resin Cements

**DOI:** 10.3390/polym14173649

**Published:** 2022-09-02

**Authors:** Andreja Carek, Ksenija Dukaric, Helena Miler, Danijela Marovic, Zrinka Tarle, Matej Par

**Affiliations:** 1 Department of Fixed Prosthodontics, School of Dental Medicine, University of Zagreb, Gunduliceva 5, 10000 Zagreb, Croatia; 2 School of Dental Medicine, University of Zagreb, Gunduliceva 5, 10000 Zagreb, Croatia; 3 Department of Endodontics and Restorative Dentistry, School of Dental Medicine, University of Zagreb, Gunduliceva 5, 10000 Zagreb, Croatia

**Keywords:** resin cements, dual-curing, light-curing, self-curing, adhesive luting, degree of conversion, flexural strength, flexural modulus

## Abstract

This study investigated the effect of different curing conditions on the degree of conversion and mechanical properties of contemporary dual-curing resin cements. The material specimens were either light-cured directly, light-cured through a 1-mm lithium disilicate glass-ceramic layer, or self-cured. The degree of conversion was measured in 0.1-mm films using Fourier-transform infrared spectroscopy 1 day, 7 days, and 28 days post-cure. Specimens used to study the flexural strength and modulus were prepared according to the ISO 4049 protocol, stored for 28 days post-cure, and subjected to accelerated aging by absolute ethanol immersion. The degree of conversion values ranged between 44.3–77.8%. Flexural strength varied between 11.4–111.1 MPa, while flexural modulus amounted to 0.7–5.5 GPa. The degree of conversion was significantly affected by material type, curing conditions, and post-cure time; however, variations in curing conditions were the least influential factor. A statistically significant effect of curing conditions on the degree of conversion was identified for only one of the five materials tested, whereas the flexural strength and modulus of all tested materials were significantly reduced in the experimental groups that were light-cured through a ceramic layer or self-cured. The effect size analysis showed that mechanical properties were most affected by the material type, while the differences in curing conditions were less influential. A comparison of the degree of conversion and mechanical properties indicated that different curing conditions may lead to significantly different flexural strength and modulus, which are not necessarily accompanied by identifiable variations in the degree of conversion.

## 1. Introduction

Resin-based cements are widely used nowadays for luting various indirect dental restorations. The basic composition of these materials is similar to that of restorative methacrylate-based, glass-filled resin composites, albeit with some adjustments which are necessary to achieve the required viscosity for thin-layer application, adhesion-promoting compounds (silanes and 10-methacryloyloxydecyl dihydrogen phosphate) and various combinations of self-cure and photo-cure initiators (camphorquinone, tertiary amines, and peroxides) [1]. Dual-curing resin-based cements are clinically preferred to purely light-curing cements because the former can better tolerate inadequate light exposure at sites that do not allow optimal access to curing light due to the morphology of the indirect restoration. Compared to light-cured, resin-based materials, the free radical-mediated polymerization of dual-cured materials is more complex, as two initiation reactions occur simultaneously and interact with each other [2]. Recognition of this complexity has led to numerous studies on dual-curing resin cements; this research has attempted to compare the effects of various curing conditions, ranging from self-curing in the dark and relying only on chemically initiated polymerization, to irradiating the material through various translucent materials, resulting in only partial activation of the photoinitiators with the expectation that chemical activation will compensate for the lack of light exposure [3,4,5,6,7,8]. These experimental conditions mimic clinical situations in which indirect restoration results in high translucency at the thinnest points, intermediate translucency at the thicker parts, or completely blocking of the curing light at certain sites [6]. Highly heterogeneous results in terms of mechanical properties have been reported by these studies, reflecting the fact that the final properties of dual-cure resin cements are markedly dependent on the material composition and experimental conditions. For example, it was reported that significant differences in flexural strength of self-adhesive dual-curing resin cements were evident immediately after curing but disappeared when the materials were aged at 37 °C for 30 days, which was attributed to the post-cure polymerization and plasticization by water [9]. Ilie et al. reported that the material composition (especially filler load) had the strongest influence on the mechanical properties, whereas the curing conditions were comparatively less important [10]. Hofmann et al. pointed out the importance of sufficient light exposure, as flexural strength, modulus, and Vicker hardness of self-cured materials were only 69–86%, 59–95%, and 86–101%, respectively, of the values obtained with dual-curing [3]. Saskalauskaite et al. reported that light-curing resulted in improved flexural strength and elastic modulus for all conventional and self-adhesive dual-curing cements tested in their study [11]. They also pointed out remarkable inter-material differences and concluded that self-adhesive composite cements cannot be considered a homogenous group of materials. Duymus et al. reported that flexural strength increased significantly when specimens were stored in distilled water for 30 days [12], while Braga et al. reported that polymerization conditions did not affect flexural strength and modulus but only hardness [13]. The above considerations lead to the general conclusion that the properties of dual-cured composite cements are a highly complex result of multiple variables related to both material composition and polymerization conditions, which are not yet fully understood.

In addition to mechanical properties, which represent macroscopic material behavior, a more fundamental parameter used to quantify the extent of polymerization in methacrylate resin-based materials is the degree of conversion (DC), defined as the fraction of C=C double bonds that have been converted into single bonds [14]. Besides being an important determinant of mechanical properties, DC also affects the biocompatibility of the material, as a higher converted material provides less residual monomer, which can be leached and cause allergic or toxic reactions [15]. Previous studies on the effects of different curing protocols on DC of dual-cured composite cements mostly agree that insufficient exposure to light (or no exposure at all in self-curing mode) results in inferior DC [4,5,6,8,16]. However, there is also evidence that low-intensity light-curing achieved through 4-mm-thick ceramic can reduce DC compared with self-curing, which has been attributed to competition between light- and chemically-induced polymerization [17]. Therefore, even a fundamental chemical parameter such as DC appears to be affected in complex and unpredictable ways. The clinical implications of the effect of curing conditions on the final properties of dual-cured resin cements are further complicated by the fact that polymerization of multifunctional methacrylates typically exhibits very high initial reaction rates followed by slow but sustained polymerization that is detectable for up to a month after onset. This suggests that the development of DC-related properties continues for some time after the prosthodontic treatment is completed. The post-cure development of DC and mechanical properties has not been sufficiently appreciated in research on dual-cure resin cements, as the majority of studies have evaluated DC and mechanical properties within less than 1 day [4,5,16,18,19,20,21] after 1 day [22,23,24,25], 3 days [26], 7 days [27,28,29,30]. Longer-term evaluations are scarce and limited to one article reporting a study conducted 14 days post-cure [31] and one conference abstract examining DC 28 days post-cure [32]. Therefore, the present study aimed to investigate the long-term development of DC over 28 days in contemporary dual-curing composite cements as a function of different curing conditions (no light exposure, light exposure through a ceramic layer, or direct unobstructed light exposure). Additionally, the mechanical properties (flexural strength and elastic modulus) were evaluated after the 28-day period, when it was assumed that the materials had reached their final properties because post-curing had been completed.

The null hypotheses were as follows:

1. The DC values and the post-cure DC development over 28 days would not be affected by the curing conditions (direct light-curing, light-curing through ceramics, and self-curing);
2. The mechanical properties measured after 28 days would not be affected by the curing conditions.

## 2. Materials and Methods

### 2.1. Composite Cements and Curing Protocols

This study investigated four dual-cure resin cements, with one light-cure resin cement as a reference. Compositional details of resin cements, as provided by the respective manufacturers, are presented in Table 1.

To evaluate the effects of different radiant exposures, cement specimens were cured using the following three protocols (except for the light-cure cement which was cured only using the first two protocols):

- LC-dir (Light-cured directly): 1080 mW/cm^2^ for 20 s by positioning the curing unit light guide tip immediately adjacent to the cement specimen;
- LC-cer (Light-cured through a ceramic layer): 280 mW/cm^2^ for 20 s due to interposition of a 1-mm layer of lithium disilicate glass-ceramic (IPS e.max Press, shade A2; Ivoclar, Schaan, Liechtenstein) between the curing unit light guide tip and the cement specimen;
- SC (Self-cured): without light illumination.

Light-curing in the LC-dir and LC-cer protocols was performed using a third-generation wide-range LED curing unit Bluephase PowerCure (Ivoclar, Schaan, Liechtenstein). The radiant exitances specified above were measured using a National Institute of Standards and Technology (NIST)-referenced and calibrated spectrometer MARC (BlueLight Analytics Inc., Halifax, NS, Canada).

### 2.2. Degree of Conversion

Thin discs of resin cement (thickness = 0.1 mm, diameter = 6 mm) were prepared by placing cements between two polyethylene terephthalate (PET) foils (Coltene, Altstätten, Switzerland) with microscope cover slides as spacers. Gentle pressure (200 g) was applied on the upper PET foil to standardize specimen thickness. The specimens were then cured using one of the above-described curing protocols. To evaluate the development of DC as a function of time, three separate subsets of specimens (*n* = 8) were prepared and left to age under dry conditions in a laboratory incubator at 37 °C for the following time points: 1 day, 7 days, and 28 days.

After being stored for a particular time, the specimens were withdrawn from the incubator and used for DC measurements employing attenuated total reflectance Fourier-transform infrared spectroscopy (ATR-FTIR). The specimen surface opposite to that exposed to the curing unit tip was pressed against the diamond ATR accessory of the FTIR spectrometer (iS50; Thermo Fisher, Madison, WI, USA). FTIR spectra were collected in absorbance mode, using 30 scans and a spectral resolution of 4 cm^−1^. In addition to the spectra of cured composite specimens, the spectra of uncured composites (*n* = 5) were recorded for DC calculation. These spectra were recorded within 20 s after extruding the uncured cement onto the ATR crystal in order to prevent artificially reduced concentrations of double C=C bonds due to self-curing. DC was calculated from the change in peak heights of the spectral band at 1638 cm^−1^ (aliphatic C=C) in the cured and uncured specimens. Since the commonly used spectral band at 1608 cm^−1^ (aromatic C⋯C) was missing in some cements, the spectral band at 1600 cm^−1^ (C-H stretching) was selected as an internal reference used to normalize FTIR spectra. The DC was calculated according to the following equation:

(1) $$\mathrm{DC}\;\left(\%\right)=\left[1-\frac{{\left(1638\;{\mathrm{cm}}^{-1}/1600\;\;{\mathrm{cm}}^{-1}\right)}_{cured}\;}{{\left(1638\;{\mathrm{cm}}^{-1}/1600\;{\mathrm{cm}}^{-1}\right)}_{uncured}}\right]\times 100$$

### 2.3. Flexural Strength and Modulus

The specimens for the three-point bending test (2 × 2 × 16 mm^3^) were prepared according to the modified ISO/DIN 4049:1998 protocol. The specimen length and inter-support span (16 mm/12 mm) were shorter compared to the most recent ISO 4049:2019 protocol (25 mm/20 mm) as the former protocol results in a smaller volume of material under load, which was considered more clinically relevant [33,34]. Uncured composite cements were applied into custom-made silicone molds, covered with PET foil, and pressed with a thick (1 mm) glass slide to extrude excess material. After removing the glass slide, the cements were cured through the PET foil using one of the above-described curing protocols. As the sample preparation according to the ISO 4049 protocol involves three overlapping light-curing as a means to compensate for the smaller diameter of the curing unit tip (9 mm) compared to the specimen length (16 mm), care was taken not to overlap the irradiated parts of the specimen for more than 1 mm. A departure from the conventional ISO 4049 specimen preparation which describes light-curing on the opposite side of the specimen, light-curing in this study was performed only from one side, in order to avoid reaching the DC plateau due to over-exposure, which would likely mask the differences between curing protocols. After curing, the specimens were lightly ground using a P4000 silicon carbide paper to remove overhangs and obtain smooth edges. The specimens were subjected to artificial aging using 28-day immersion in distilled water at 37 °C followed by 7-day immersion in absolute ethanol (Merck, Darmstadt, Germany) [35]. Twenty specimens were prepared per experimental group (*n* = 20). Mechanical testing was performed in a universal testing machine (Inspekt Duo 5kN-M; Hegewald & Peschke, Nossen, Germany) using an inter-support span of 12 mm and a crosshead speed of 0.5 mm/min according to NIST 4877 guidelines. Specimens were immersed in distilled water at room temperature during the testing procedure. Flexural strength (FS) and flexural modulus (FM) were calculated according to the following equations:

(2) $$\mathrm{FS}=\frac{3F_{f}l}{2bh^{2}}$$

(3) $$\mathrm{FM}=\frac{F_{l}l^{3}}{4bh^{3}y_{l}}$$

where *F_f_* = force at fracture (N), *l* = inter-support span (mm), *b* = specimen width (mm), *h* = specimen height (mm), *F_l_* = force at the end of the linear part of the force-deflection diagram (N), and *y_l_* = deflection at the end of the linear part of the force-deflection diagram (mm).

### 2.4. Statistical Analysis

The assumption of a normal distribution of data was verified using Shapiro-Wilk’s test and the inspection of normal Q-Q diagrams. The FS and FM data were analyzed using a three-way ANOVA with factors “material”, “curing protocol”, and “post-cure time”, whereas for the comparison of the DC data, a two-way ANOVA with factors “material” and “curing protocol” was used. Since statistically significant results of the *omnibus* tests and significant interactions among the factors were identified, the analysis was followed by one-way ANOVAs to explore the effects of individual factors at each level of the remaining factors. Tukey post-hoc adjustment was used for multiple comparisons. Statistical analysis was performed using SPSS 25 (IBM, Armonk, NY, USA) at an overall significance level of 0.05.

## 3. Results

Table 2 shows that all factors in a full factorial model, i.e., “material”, “curing protocol”, and “post-cure time”, exerted statistically significant effects on DC. The binary interactions that involved the factor “material” were statistically significant, indicating different responses to curing protocols and post-cure DC development among different materials. In contrast, the interaction between the factors “curing protocol” and “post-cure time” was not statistically significant, indicating a similar post-cure DC development for all curing protocols. Partial eta-squared values indicate the following relative effect size for individual factors on DC: post-cure time > material > curing protocol.

Statistically significant effects of the factors “material” and “curing protocol” and their interaction were also identified for FS and FM, as shown in Table 3. For both FS and FM, a higher effect size was observed for the factor “material” compared to “curing protocol”.

**Figure 1:**
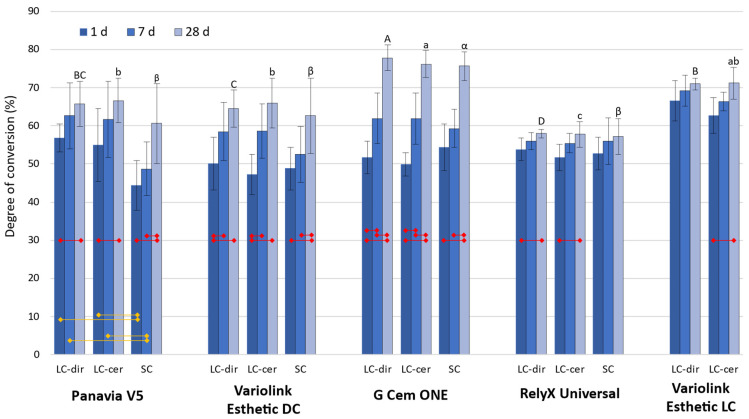
Degree of conversion (mean values ± 1 standard deviation) measured after 1 day, 7 days, and 28 days for the following curing protocols: LC-dir—light-cured directly, LC-cer—light-cured through a ceramic layer, SC—self-cured. Yellow lines at the bottom of the plot denote statistically significant differences among curing protocols. Red lines in the middle of the plot denote statistically significant differences among time points. Same letters above the bars indicate statistically similar values for inter-material comparisons of the degree of conversion values reached 28 days post-cure. These DC values were compared for each of three curing protocols separately, the comparisons within individual curing protocols (LC-dir, LC-cer, and SC) are represented by uppercase, lowercase, and Greek letters, respectively.

The DC values shown in Figure 1 ranged between 44.3–77.8%. Statistically significant post-cure DC increase was identified for all combinations of materials and curing protocols, except for RelyX Universal (SC protocol) and Variolink Esthetic LC (LC-dir protocol). The extent of DC increase between 1 day and 28 days differed considerably among the materials, ranging from 4.1–6.1% for RelyX Universal to 21.2–26.2% for G Cem One. The effect of curing protocols on DC was identified only for Panavia V5, for which the SC protocol after 1 day and 7 days resulted in significantly lower DC compared to LC-dir and LC-cer. However, after 28 days, the DC values for Panavia V5 became statistically similar among all curing protocols. The inter-material comparisons showed statistically significant differences with the highest values observed for G Cem One and lowest values for RelyX Universal.

**Figure 2:**
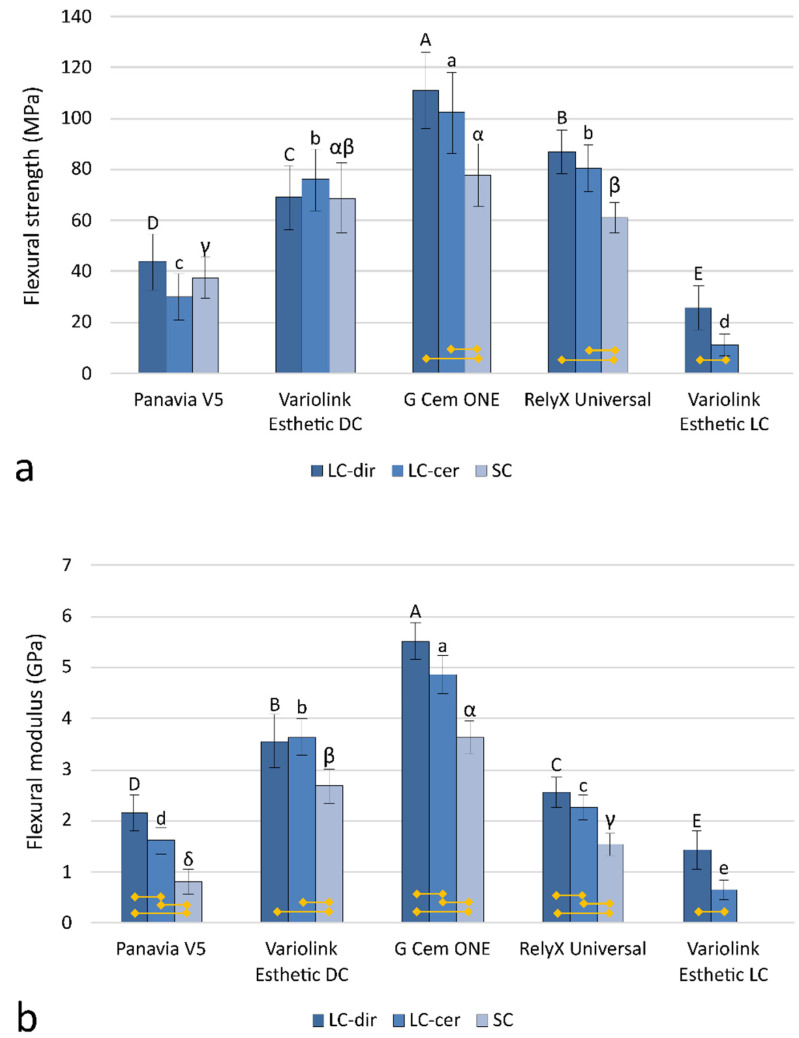
Flexural strength (**a**) and modulus (**b**) represented as mean values ± 1 standard deviation, measured after 28 days in distilled water followed by artificial aging via 7-day immersion in absolute ethanol for the following curing protocols: LC-dir—light-cured directly, LC-cer—light-cured through a ceramic layer, SC—self-cured. Yellow lines at the bottom of the plot denote statistically significant differences among curing protocols. The results of inter-material comparisons within individual curing protocols (LC-dir, LC-cer, and SC) are represented by uppercase, lowercase, and Greek letters, respectively.

The mechanical properties in Figure 2 varied between 11.4–111.1 MPa (FS) and 0.7–5.5 GPa (FM). Two out of five materials showed no significant effects of curing protocols on FS (Panavia V5 and Variolink Esthetic DC), whereas in other materials a statistically significant FS reduction among the curing protocols (LC-dir > LC-cer > SC) was identified. FS results were more discriminative among curing protocols, showing statistically significant differences for all five materials with the same ranking as found for FS (LC-dir > LC-cer > SC). The inter-material differences in FS and FM were statistically significant differences and more extensive than the differences among curing protocols within each individual material.

## 4. Discussion

This study evaluated the effect of different curing protocols on the DC and mechanical properties of contemporary dual-curing resin cements. To evaluate the post-cure DC development, measurements were performed after 1, 7, and 28 days. Since DC increased significantly during the 28-day observation period, the specimens for FS and FM evaluation were given 28 days to complete the post-cure polymerization before being immersed in ethanol as a means of accelerated aging. A statistically significant effect of curing protocols on DC was identified for only one of the five materials tested (Panavia V5). In contrast to these results, the mechanical properties of all tested materials were significantly reduced in the experimental groups that were light-cured through a ceramic layer or self-cured. Hence, the first null hypothesis was rejected only for Panavia V5, while the second null hypothesis was rejected for all tested materials.

Dual-curing cements are characterized by two simultaneously occurring modes for initiating free radical-mediated dimethacrylate polymerization. The light-curing mode is intended for rapid on-demand setting, while the self-curing mode is intended to compensate for the lack of light exposure at sites that are covered by opaque portions of the indirect restoration and hence inaccessible to illumination [2]. Both curing modes trigger the same chain reaction which then propagates until free radicals are terminated or the viscosity of the material is increased to the point where diffusion limitations stop the polymerization [36]. Partly disagreeing with other studies which reported a significant effect of curing conditions on DC for most dual-curing resin cements [4,5,6,8,16,17], our results showed that after 28 days, all curing protocols resulted in comparable DC values within a given cement, regardless of the curing protocol used.

Significantly better DC attained by direct light-curing compared to curing through ceramics or self-curing was identified only for Panavia V5 after 1 day and 7 days, but the differences leveled off after 28 days and became statistically similar for all curing protocols. The manufacturer’s information for Panavia V5 indicates that this material does not contain the usual amine co-initiator in order to improve the color stability of the material. Since the chemical composition of the co-initiator that replaced amine has not been disclosed by the manufacturer, we could not speculate whether it is a possible cause for the different polymerization behavior of Panavia V5 compared to the other materials tested. A possible reason for the lack of statistically significant effects of curing conditions on DC in four out of five tested materials could also be the high scatter of data in some experimental groups (coefficients of variability ranging from 2.0–17.3%), which is due to the use of thin films sensitive to polymerization inhibition by atmospheric oxygen [37].

The slow development of DC over a certain post-cure period is a well-known phenomenon in dimethacrylate-based dental resin composites, explained by the immobilization of reactive species within the polymerizing network [38]. Most of the polymerization reaction takes place within a few seconds to minutes after the onset of curing, i.e., while the reaction medium is still sufficiently mobile to allow unreacted monomer molecules to approach the reactive chain ends of the growing polymer radicals, thus propagating the reaction. As polymerization proceeds and the polymer network becomes vitrified, the mobility of the reactive species is severely impaired and the polymerization continues at an increasingly slower rate over several days or weeks with its kinetics governed by diffusion limitations [39]. The extent of post-cure polymerization depends on several factors, which essentially determine the mobility of the network and the amount of monomer after completion of the initial fast part of polymerization [40]. Depending on the material properties and experimental conditions, some studies on resin cements reported that DC does not increase beyond 24 h [28], while others indicated that polymerization can last up to 7 days [30] or 28 days [32]. The increase of DC over 28 days after curing observed in the present study suggests that the final mechanical properties and biocompatibility of the fully cured material are not achieved immediately after restoration cementation and patient release but rather develop gradually. In addition, the very different extents of post-cure polymerization observed for different materials indicate that some cements reach their final properties more readily (e.g., RelyX Universal with a 28-day post-cure DC increase of only 4.1–6.1%), while for other materials the properties develop more gradually (e.g., G Cem One with a 28-day post-cure DC increase of 21.2–26.2%). Such extensive post-cure polymerization in G Cem One could be attributed to its highest filler among all tested cements (70 wt%). It is known that a high filler content in resin composite materials contributes to earlier immobilization, resulting in a lower initial DC, which in turn leads to a more extensive post-cure DC increase [41]. Clinically, the most suitable cement would be the one that reaches its final DC as soon as possible after application, as this means not only that mechanical durability is achieved earlier but also that less time and a smaller amount of potentially toxic monomers are available for diffusion into the pulp tissue [42,43]. In this context, the comparatively slower and more extensive post-cure polymerization in G Cem One appears to be an unfavorable material property. On the other hand, the same factor is considered responsible for the more extensive post-cure reaction in G Cem One, namely the high filler load, resulted in the significantly highest mechanical properties of this material among all other materials tested. This underlines the fact that a compositional variable can improve certain material properties, but the improvement may be at the expense of another clinically relevant property.

Considering the very different extents of post-cure DC increase among the materials tested, it should be noted that the DC values reached within 24 h, which are evaluated in the vast majority of studies on dual-curing cements [4,5,16,18,19,20,21], are not necessarily predictive of the DC values to be attained after fully completing the post-cure polymerization. These values may be useful as an indicator of intermediate properties while the polymerization is still in progress. However, longer post-cure times are advisable for evaluating the final material properties.

DC values obtained by vibrational spectroscopy have been reported to vary due to differences in methodology, specimen preparation, and material composition [44], which precludes direct comparability of DC results with data reported in other studies. The inability of various spectroscopic setups to produce consistent DC values stems from the fact that, in common practice, a linear relationship between the concentration of C=C double bonds and the intensity of the spectral band at 1638 cm^−1^ is assumed, without calibration using external references. Since there is no guarantee of the linearity of this relationship, which is likely to be affected by the composition of individual materials, small inter-material differences in DC do not necessarily mean that one material has better conversion than another. On the other hand, differences among DC values attained by different curing modes within a single material are more meaningful because the comparisons within a material exclude the possible influence of material composition.

For a given resin cement, the variations of DC are the main determinant of the material mechanical properties of the material, i.e., higher conversion is generally associated with better mechanical properties [4,45]. However, deviations from this relationship are known to occur, mainly because DC reflects only the percentage of double bonds converted but does not provide further information about the structure of the polymer network [46]. In general, faster polymerization with more polymeric chain growth centers leads to better interconnected networks with improved mechanical properties. Conversely, slower polymerization results in less crosslinked and more linear polymer chains that are mechanically weaker [47]. This has been reported for restorative resin composites, where large differences in curing light intensity can lead to different network structures [48]. For dual-curing resin cements, the differences are even more pronounced because their chemical initiator system provides a slow but constant initiation rate in the self-curing mode, whereas the initiation rate in the light-curing mode varies greatly depending on the intensity of the curing light at the particular site [4,16]. Therefore, DC alone cannot reliably predict the mechanical properties of materials polymerized at widely varying rates, such as dual-curing resin cements. This consideration is supported by the results of the present study, as the mechanical properties obtained with different curing protocols varied significantly, although the curing protocols had no statistically significant effect on DC after 28 days. For G Cem One and RelyX Universal, self-curing led to FS values that were 30% lower than the maximum values achieved by direct light curing. FM was reduced even more by self-curing, with reductions of 25–62% being statistically significant for all dual-cure materials tested. The differences in mechanical properties due to curing through ceramics were also observed for the light-cured material Variolink Esthetic LC, which had 56% lower FS and 55% lower FM compared to direct light-curing.

The observed differences in mechanical properties seem surprising considering that the DC data after 28 days showed no significant differences among curing protocols. However, these pronounced differences among curing modes can be explained by the aggressive accelerated aging protocol (7-day immersion in absolute ethanol). This type of accelerated aging is capable of highlighting subtle differences in network structure and is therefore commonly used to indirectly quantify crosslink density in methacrylate resin-based composites [49]. Ethanol penetrates the interstices between the chains, swelling and plasticizing the polymeric network. The measured mechanical properties were significantly affected by these structural differences in the polymer network. On the other hand, the differences were not identifiable by the DC measurements, which only quantify C=C bond consumption but are not sensitive to polymer network structure. The aggressive accelerated aging protocol also explains the fact that FS and FM measured in the present study were lower than those reported for resin cements in other studies which commonly used less aggressive water storage [3,11,12,13]. The highly pronounced inter-material differences may also be attributed to aggressive aging. Although artificial aging in absolute ethanol was beneficial in highlighting inter-material differences in mechanical properties, it should be noted that such a worst-case scenario of material degradation does not occur regularly in the oral cavity, hence the large differences in performance among the tested materials may not be present clinically. Furthermore, the absolute FS and FM values measured after aggressive accelerated aging only show the relative performance of the materials and cannot be directly related to the probability of clinical success or failure.

The specimens used for the mechanical tests were artificially aged by immersion in water and ethanol, as the properties of composite materials are known to degrade when exposed to an aqueous medium [50]. Since this degradation occurs over many years of the service life of the restoration, artificial accelerated aging methods are often used, the most common being thermocycling between 5–55 °C [51] and immersion in absolute ethanol [52]. Despite its popularity in dental materials research, thermocycling was not used in the present study because the temperature of 55 °C in the “hot” part of the cycle could artificially increase DC by enhancing the mobility of reactive species [53], thus overestimating the material properties regularly obtained by post-curing the material in the oral cavity at temperatures around 37 °C. A more suitable method of artificial aging was chosen, involving storage at 37 °C in distilled water for 28 days followed by immersion in absolute ethanol for 7 days [54,55]. Since post-cure polymerization was more pronounced in some materials than in others, the 28-day aging period before immersion in absolute ethanol was important to avoid underestimating the mechanical properties of the materials with more extensive post-cure polymerization.

ISO 4049 prescribes a minimum FS of 50 MPa for luting materials. Due to the aggressive artificial aging used in our study, our results should not be directly compared to these reference values. However, the fact that some of the tested resin cements (Variolink Esthetic DC, G Cem One, and RelyX Universal) maintained their FS above the ISO 4049 threshold despite being subjected to aggressive aging suggests that these materials would likely meet the prescribed value of 50 MPa even if subjected to much less aggressive aging as prescribed in the ISO 4049 protocol, i.e., one day in distilled water at body temperature.

Heterogeneity of mechanical properties, as observed in the present study, also occurs in clinical scenarios, because when complex indirect restorations are cemented using dual-cure resin cements, the curing light is considerably attenuated or completely blocked at some sites. Hence, different gradients of mechanical properties occur at the interface between the restoration and the dental hard tissues. These gradients may not be as pronounced as the differences resulting from an aggressive accelerated aging protocol in this study, or they may take some time to develop as the material ages.

## 5. Conclusions

The 28-day development of DC and the response to different curing conditions proved to be highly material-dependent. Most of the investigated cements showed a significant post-cure DC increase, whereas variations in curing conditions were less influential for the final DC values. 

The comparison of DC and mechanical properties showed that different curing conditions may lead to significantly different FS and FM, which are not necessarily accompanied by different DC values. Direct light-curing led to the highest FS and FM values, suggesting that the investigated dual-cure cements should be optimally light-cured in order to maximize mechanical properties.

## Figures and Tables

**Table 1:** Composition of the investigated resin cements as provided by their respective manufacturers.

Material Type	Material Name	Manufacturer	Composition	Filler Load	Shade/ LOT No.
Dual-cure resin cement	Panavia V5	Kuraray Noritake, Tokyo, Japan	Bisphenol A diglycidylmethacrylate, triethyleneglycol dimethacrylate, hydrophobic aromatic dimethacrylate, hydrophilic aliphatic dimethacrylate, initiators, accelerators, silanated barium glass, silanated alminium oxide, silanated fluoroalminosilicate glass, colloidal silica, camphorquinone, pigments	61 wt%/ 38 vol%	Clear/6P0044
Dual-cure resin cement	Variolink Esthetic DC	Ivoclar, Schaan, Liechtenstein	Urethane dimethacrylate, other methacrylate monomers, are ytterbium trifluoride, spheroid mixed oxide, initiators, stabilizers, pigments	38 vol%	Neutral/Z029SK
Self-adhesive dual-cure resin cement	G-Cem ONE	GC, Tokyo, Japan	Fluoro-alumino-silicate-glass, urethane dimethacrylate, dimethacrylate, phosphoric ester monomer, silicone dioxide, initiators	70 wt% *	Translucent/2106211
Self-adhesive dual-cure resin cement	RelyX Universal	3M, St. Paul, MN, USA	Bisphenol-A derivative free dimethacrylate monomers, phosphorylated dimethacrylate adhesion monomers, photoinitiator system, novel amphiphilic redox initiator system, radiopaque fillers and rheological additives, pigments	52 wt%	TR/8275139
Light-cure resin cement	Variolink Esthetic LC	Ivoclar, Schaan, Liechtenstein	Urethane dimethacrylate, other methacrylate monomers, are ytterbium trifluoride, spheroid mixed oxide, initiators, stabilizers, pigments	38 vol%	Neutral/Z023VY

* Approximate value according to personal communication with manufacturer’s representative.

**Table 2:** Statistical significance (*p*-values) and practical significance (partial eta-squared values) of the factors “material”, “curing protocol”, and “post-cure time” for degree of conversion.

Factor	*p*-Value	Partial Eta-Squared
**Material**	<0.001	0.350
**Curing protocol**	<0.001	0.072
**Post-cure time**	<0.001	0.480
**Material × Curing protocol**	<0.001	0.102
**Material × Time post-cure**	<0.001	0.230
**Curing protocol × Time post-cure**	0.16	N/A
**Material × Curing protocol × Post-cure time**	0.88	N/A

**Table 3:** Statistical significance (*p*-values) and practical significance (partial eta-squared values) of the factors “material” and “curing protocol” for flexural strength and flexural modulus.

	Factor	*p*-Value	Partial Eta-Squared
**Flexural** **strength**	Material	<0.001	0.858
	Curing protocol	<0.001	0.268
	Material × Curing protocol	<0.001	0.245
**Flexural** **modulus**	Material	<0.001	0.938
	Curing protocol	<0.001	0.700
	Material × Curing protocol	<0.001	0.213

## Data Availability

Not applicable.
